# Point-of-Care Diagnosis of Malaria Using a Simple, Purification-Free DNA Extraction Method Coupled with Loop-Mediated Isothermal Amplification-Lateral Flow

**DOI:** 10.3390/tropicalmed8040199

**Published:** 2023-03-29

**Authors:** Meng Yee Lai, Lee Phone Youth Zen, Mohd Hafizi Abdul Hamid, Jenarun Jelip, Rose Nani Mudin, Vun Jan Shui Ivan, Lee Ngie Ping Francis, Izreena Saihidi, Yee Ling Lau

**Affiliations:** 1 Department of Parasitology, Faculty of Medicine, Universiti Malaya, Kuala Lumpur 50603, Malaysia; mengylai11@um.edu.my (M.Y.L.); zen1py@hotmail.com (L.P.Y.Z.); 2 Vector Borne Disease Sector, Ministry of Health, Putrajaya 62590, Malaysia; dr.mhafizi@moh.gov.my (M.H.A.H.); jenarun@moh.gov.my (J.J.); drrose@moh.gov.my (R.N.M.); 3 Hospital Kapit, Pathology Unit, Jalan Mamora, Kapit 96800, Malaysia; ivanvun@gmail.com (V.J.S.I.); aloysiusfrancislee@gmail.com (L.N.P.F.); izreena88@moh.gov.my (I.S.)

**Keywords:** malaria, DNA extraction, LAMP, molecular diagnosis, filter paper-based, lateral flow, point-of-care

## Abstract

We propose a protocol suitable for point-of-care diagnosis of malaria utilizing a simple and purification-free DNA extraction method with the combination of loop-mediated isothermal amplification assay and lateral flow (LAMP-LF). The multiplex LAMP-LF platform developed here can simultaneously detect *Plasmodium knowlesi, P. vivax, P. falciparum,* and *Plasmodium* genus (for *P. malariae* and *P. ovale*). Through the capillary effect, the results can be observed by the red band signal on the test and control lines within 5 min. The developed multiplex LAMP-LF was tested with 86 clinical blood samples on-site at Hospital Kapit, Sarawak, Malaysia. By using microscopy as the reference method, the multiplex LAMP-LF showed 100% sensitivity (95% confidence interval (CI): 91.4 to 100.00%) and 97.8% specificity (95% CI: 88.2% to 99.9%). The high sensitivity and specificity of multiplex LAMP-LF make it ideal for use as a point-of-care diagnostic tool. The simple and purification-free DNA extraction protocol can be employed as an alternative DNA extraction method for malaria diagnosis in resource-limited settings. By combining the simple DNA extraction protocol and multiplex LAMP-LF approach, we aim to develop a simple-to-handle and easy-to-read molecular diagnostic tool for malaria in both laboratory and on-site settings.

## 1. Introduction

Malaria is one of the most severe public health problems globally. In 2021, there were an estimated 247 million malaria cases worldwide, which increased from 245 million cases in 2020 [1]. To effectively manage and control malaria, the development of a rapid molecular diagnostic tool for malaria is an urgent need. To date, there are five *Plasmodium* species that are known to infect humans, namely *P. falciparum, P. vivax, P. malariae*, *P. ovale,* and *P. knowlesi*.

Typically, microscopy, rapid diagnostic tests, and/or molecular techniques are used to diagnose malaria. Microscopy continues to be the gold standard for the laboratory confirmation of malaria, according to the United States Center for Disease Control. However, to correctly identify and distinguish the *Plasmodium* species, microscopy needs a high level of competency. Molecular approaches are frequently used in diagnosis due to their adaptability and high sensitivity, and species-specific primers can identify various *Plasmodium* species. Nested polymerase chain reaction (PCR) is always used as a reference standard for the molecular diagnosis of malaria. However, it requires a thermocycler and takes 4–6 h to complete a reaction. Thus, these disadvantages hinder its use as a molecular diagnostic tool in resource-poor and non-laboratory settings.

To circumvent the drawbacks associated with nested PCR, isothermal amplification techniques have been developed as nucleic acid-based detection tools. Among all the isothermal techniques [2,3,4,5,6,7,8,9], the loop-mediated isothermal amplification (LAMP) method has been the most popular nucleic acid amplification technique. LAMP diagnosis of malaria is fast (turnaround time less than 1 h) and has a better sensitivity detection (as low as 0.5 to 0.05 parasites/µL) than PCR [10]. LAMP products can be analyzed by various methods such as agarose gel electrophoresis, using fluorescent, metal ion indicator, and pH-sensitive dyes as well as visualization of turbidity [11,12,13,14].

LAMP products can also be detected by using lateral flow-based methods. Lateral flow is a well-known paper-based platform for the detection of analytes (with specifically labeled haptens) using an antibody-antigen capture mechanism. Haptens that are commonly used in primer labeling include fluorescein, biotin, dinitrophenol, and digoxigenin. The combination of LAMP and lateral flow (LAMP-LF) assay has been used for the detection of various pathogens such as *P. falciparum* [10], *Toxoplasma gondii* [15], *Mycoplasma ovipneumoniae* [14], *Babesia bovis*, *Babesia bigemina* [16], African trypanosome [17], *Mycobacterium tuberculosis* [18], and *Brucella* spp. [19]. In this study, we developed a multiplex LAMP approach coupled with LF for the simultaneous detection of 5 *Plasmodium* species.

Furthermore, we utilized a simple and purification-free DNA extraction method that has been shown to expedite malaria diagnosis compared to commercial extraction kits that require a long incubation period and multiple purification steps [20]. With the combination of a simple DNA extraction protocol and LAMP-LF, we propose a protocol suitable for point-of-care testing on malaria that meets the World Health Organization (WHO) ASSURED criteria (affordable, sensitive, specific, user-friendly, rapid/robust, equipment-free, and deliverable) [21,22,23]. We validated our established multiplex LAMP-LF’s performance by detecting DNA extracted from clinical samples from several states in Malaysia.

The aim of our study is to develop a multiplex LAMP-LF for simultaneous detection of 5 human *Plasmodium* species. By using the simple DNA extraction protocol, the time required for extraction is reduced to ~8 min. The results read-out is performed by using custommade lateral flow strips. Incorporation of the easy-handled DNA extraction method makes multiplex LAMP-LF well-adapted to resources-limited settings.

## 2. Materials and Methods

### 2.1. Sample Collection

A total of 68 archived malaria samples were included in this study for the testing of the prototype, of which 26 were *P. knowlesi*, 9 *P. vivax*, 9 *P. falciparum*, 2 *P. ovale*, 2 *P. malariae,* and 20 healthy donor blood samples without any malaria symptoms such as fever, headache, and chills. The archived blood samples were stored in −20 °C. These blood samples were collected from district hospitals in Selangor, Kelantan, Negeri Sembilan, Pahang, and Perak, from 2019 to 2021. The parasitemia range of the archived samples was 0.02 to 1.33%.

Another sum of 86 blood samples (32 *P. knowlesi*, 7 *P. vivax*, 3 *P. falciparum* and 44 malaria negative based on microscopic examination) were collected from febrile patients with suspected malaria at a point-of-care setting. These blood samples were collected from Hospital Kapit, Sarawak in April–September 2022. The parasitemia range of clinical samples was 0.004 to 1.16%. The samples were collected on-site and tested immediately. In the case of delay testing, the samples were stored at 4 °C. These samples were also confirmed by nested PCR according to the cycling protocols as described by Snounou et al. [24] and Imwong et al. [25]. The sample size was calculated based on Hajian-Tilaki [26]. After considering a 95% confidence interval and 80% power to detect a difference of 15% from the presumption value of standard deviation (Se) = 80%, the sample size was 42. In our clinical screening, 42 malaria samples and 44 healthy blood samples were tested. This study was approved by the Medical Ethics Committee of UMMC (MEC reference no. 817.18 and 908.11) and National Medical Research Registry (reference No. NMRR-12-1105-13079). All samples were collected prior to antimalarial treatment.

### 2.2. Alternative DNA Extraction Coupled with LAMP-LF Assay

The extraction method and buffers were adapted from Zou et al. with minor modifications [20]. A total of 60 µL blood samples and 240 µL of lysis buffer (800 mM guanidine hydrochloride, 50 mM Tris (pH 8), 0.5% Triton™ X-100, 1% Tween-20, 40 µg/mL proteinase K) was added into a 1.5 mL microcentrifuge tube. The tube consisting of blood and lysis buffer mixture was constantly inverted until homogenous and transparent. In the tube consisting of the lysate mixture, a 6-mm diameter Whatman grade 1 qualitative filter paper was dipped into the mixture and incubated for 1 min. The filter paper was then removed from the blood lysis mixture and washed with 1 mL of washing buffer (10 mM Tris (pH 8.0) and 0.1% Tween-20). The filter paper was dipped into the tube containing the washing buffer for 1 min. After the washing step, the filter paper was ready for the LAMP assay by dipping it into the PCR tube 5 times and removed.

### 2.3. Preparation of 40 nm Diameter Gold Nanoparticles

The gold nanoparticles of 40 nm diameter were synthesized by the citrate reduction method as previously reported by Hermanson [27] and Yokota [28] with minor modifications. Two hundred microliters of 1% gold chloride III (Sigma-Aldrich, St. Louis, MO, USA) were added to the conical flask and topped up with 19.8 mL of distilled water. The mixture was then heated to a boiling point with a magnetic stirrer, while 200 µL of 1% sodium citrate (Sigma-Aldrich, St. Louis, MO, USA) was added. During the process of boiling and stirring, the solution changed from colorless to brilliant red in less than one minute. The heating and stirring were continued for another 5 min. The final volume of solution was topped up to 20.2 mL with distilled water and cooled in an ice bath. At this stage, the mixture was stored at 4 °C in a Schott bottle (wrapped with aluminum foil) until further use.

### 2.4. Conjugation of Streptavidin with Gold Nanoparticles

Conjugation of streptavidin (Santa Cruz, Dallas, TX, USA) with gold nanoparticles was prepared according to the methods described previously with minor modifications [27,28]. Prior to use, the pH value of gold nanoparticle solution was adjusted to 7.0 by using 0.2 M sodium carbonate and 0.1 M hydrochloric acid. Two hundred microliters of streptavidin was added to 20 mL of gold nanoparticle solution. The mixture was stirred with a magnetic stirrer for 30 min at room temperature in the dark (Schott bottle was wrapped with aluminum foil), followed by the addition of 200 µL 10% bovine serum albumin (BSA) (Sigma-Aldrich, St. Louis, MO, USA) and 800 µL of 2% polyethylene glycol 3000 (Sigma-Aldrich, St. Louis, MO, USA). The mixture was incubated for 1 h at room temperature. After that, the mixture was centrifuged for 30 min at 20,000× *g*, 4 °C. The supernatant containing unbound streptavidin was removed as much as possible. The pellet (consisting of conjugated streptavidin) was resuspended with 500 µL of 0.02% polyethylene glycol 3000, 0.05% sodium azide (Sigma-Aldrich, St. Louis, MO, USA). The gold-conjugated streptavidin was then stored at 4 °C.

### 2.5. Construction of Lateral Flow Strips

**Figure 1:**
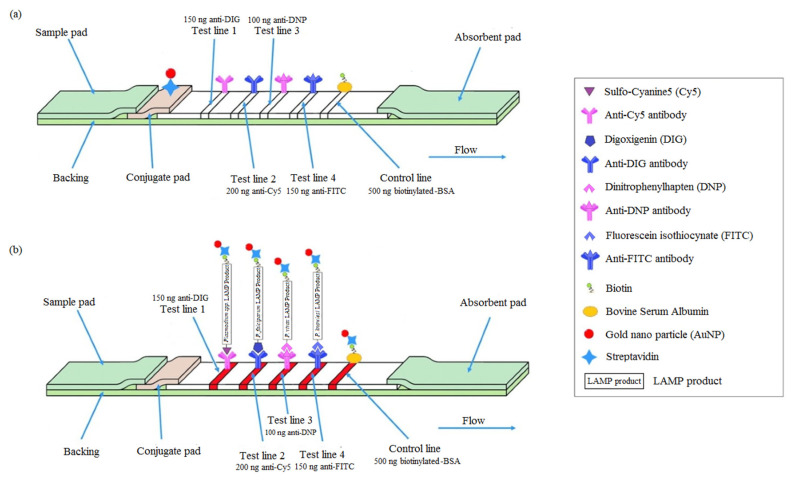

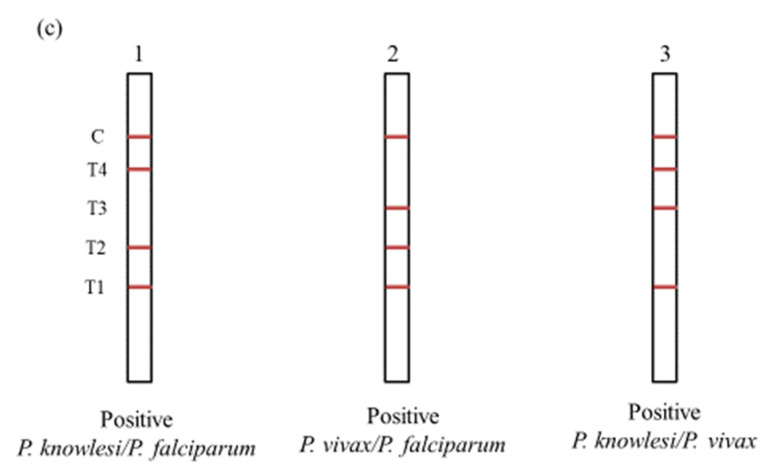
(**a**) Design of LAMP-LF strip. There were four regions on the lateral flow strip, which include a sample pad, a conjugate pad, a nitrocellulose membrane and an absorbent pad. Five antibodies were coated on the nitrocellulose membrane, which corresponds to four test lines and one control line. Test line 1 (TL1) was coated with 150 ng anti-DIG; Test line 2 (TL2) was coated with 200 ng anti-Cy5; Test line 3 (TL3) was coated with 100 ng anti-DNP; Test line 4 (TL4) was coated with 150 ng anti-FITC; The control line (CL) was coated with 500 ng biotinylated-BSA. (**b**) Detection of LAMP products on the lateral flow strip. The lateral flow strips can detect 4 targets; *Plasmodium* spp.-LAMP product, *P. falciparum*-LAMP product, *P. vivax*-LAMP product, and *P. knowlesi*-LAMP product at the respective coated test lines and control line. (**c**) Schematic representation of the lateral flow test strips used for detection of multiple infections of *Plasmodium* spp. In blood samples. Interpretation of the multiplex LAMP-LF results: 1, positive for *P. knowlesi* and *P. falciparum* mixed infections (four red lines at the readout; CL, TL4, TL2, and TL1); 2, positive for *P. falciparum* and *P. vivax* mixed infections (four red lines at the readout; CL, TL3, TL2, and TL1); 3, positive for *P. vivax* and *P. knowlesi* mixed infections (four red lines at the readout; CL, TL4, TL3, and TL1). CL: control line; TL1: test line 1; TL2: test line 2; TL3: test line 3; TL4: test line 4.

The lateral flow strips consisted of four components, that were assembled on a backing card (a sample pad, a conjugate pad, a nitrocellulose membrane and an absorbent pad) (Figure 1(A1)). Prior to immobilization, the conjugate pad was pre-treated by submersion in a solution containing 1x PBS, 1% Tween-20, 0.5% BSA and 5% sucrose for 5 s. The gold-conjugated streptavidin was immobilized on the conjugate pad and left to dry overnight in a desiccator. Next, 750 mg/mL of anti-digoxigenin (anti-DIG) (Jackson ImmunoResearch Laboratories, West Grove, PA, USA), 1000 mg/mL of anti-cyanine 5 (anti-Cy5) (Abcam, Cambridge, UK), 500 mg/mL of anti-dinitrophenol (anti-DNP) (Vector Laboratories, California, United States), 750 mg/mL of anti-fluorescein isothiocyanate (anti-FITC) (Genetex, Irvine, CA, USA), and 2500 mg/mL of biotinylated-BSA (Sigma-Aldrich, St. Louis, MO, USA) were dispensed on the nitrocellulose membrane (1 cm diameter) to form test lines 1 to 4 and a control line. The distance between the two lines was approximately 5 mm. The spotting of the test lines and control lines was performed by using BioDot XYZ3060™ Dispense Platform (BioDot, Irvine, CA, USA). The nitrocellulose membrane with the immobilized antibodies was left to dry overnight in a desiccator at room temperature. Then, the assembled lateral flow strips were cut into 2-mm dipsticks using the CM5000™ Guillotine Cutter (BioDot, Irvine, CA, USA) and dry-stored at room temperature (<20% relative humidity) with gel desiccant beads until use. The lateral flow strips produced here can detect 4 targets, *Plasmodium* spp.-LAMP product, *P. falciparum*-LAMP product, *P. vivax*-LAMP product, and *P. knowlesi*-LAMP product at the respective coated test lines (Figure 1a). Figure 1b indicates a schematic representation of the lateral flow test strips used for detection of multiple infections of *Plasmodium* spp. in blood samples.

### 2.6. LAMP Assay

The LAMP assay and primers were adapted from Lau et al. with minor modifications to the loop primers [29]. The loop primers of different species were labeled with specific hapten (biotin, DIG, Cy5, DNP, and FITC) (Table 1). Initially, the LAMP assay was performed in single reaction. The 25-μL reaction mixture consisted of 6.7 µL distilled water, 2.5 µL of 10X isothermal amplification buffer, 3.5 µL deoxynucleotide triphosphates (10 mM), 1.5 µL magnesium sulphate (100 mM), 1 μL *Bacillus stearothermophilus* (*Bst*) 2.0 WarmStart^®^ DNA Polymerase (New England Biolabs, Ipswich, MA, USA ), 1.6 μM forward inner primer (FIP) and backward inner primer (BIP), 0.8 μM forward loop primer (FLP) and backward loop primer (BLP), 0.2 μM forward primer (F3) and backward primer (B3) primer, and 0.8 M betaine (Sigma-Aldrich, St. Louis, MO, USA). The filter paper with extracted DNA was used as a template. The LAMP assay was incubated in a heat block at 65 °C for 50 min and inactivated at 80 °C for 2 min. After that, 4 μL of LAMP products were loaded onto the lateral flow strip, followed by 60 μL of 1x PBS. The result was then observed at 5 min. After the LAMP assay was confirmed working in singleplex reaction, all four sets of primers were then added in one tube for the multiplex LAMP. The preparation of the master mixture was similar as mentioned above except with the addition of all four sets of each primer, 1.6 μM FIP and BIP, 0.8 μM FLP and BLP, 0.2 μM F3 and B3. The LAMP assay was incubated in a heat block at 65 °C for 50 min and inactivated at 80 °C for 2 min. After that, 4 μL of LAMP products were loaded onto the lateral flow strip, followed by 60 μL of 1x PBS. The result was then observed at 5 min. Multiplex LAMP-LF was used for archived and fresh samples screening.

### 2.7. Clinical Sensitivity and Specificity

By using microscopy as the reference standard, the clinical sensitivity and specificity of the multiplex LAMP-LF assay were determined based on 86 whole blood samples collected on-site. Sensitivity was calculated as (number of true positives)/(number of true positives + number of false negatives), and specificity was calculated as (number of true negatives)/(number of true negatives + number of false positives). The agreement between the diagnostic test was calculated using the Kappa coefficient.

## 3. Results

**Figure 2:**
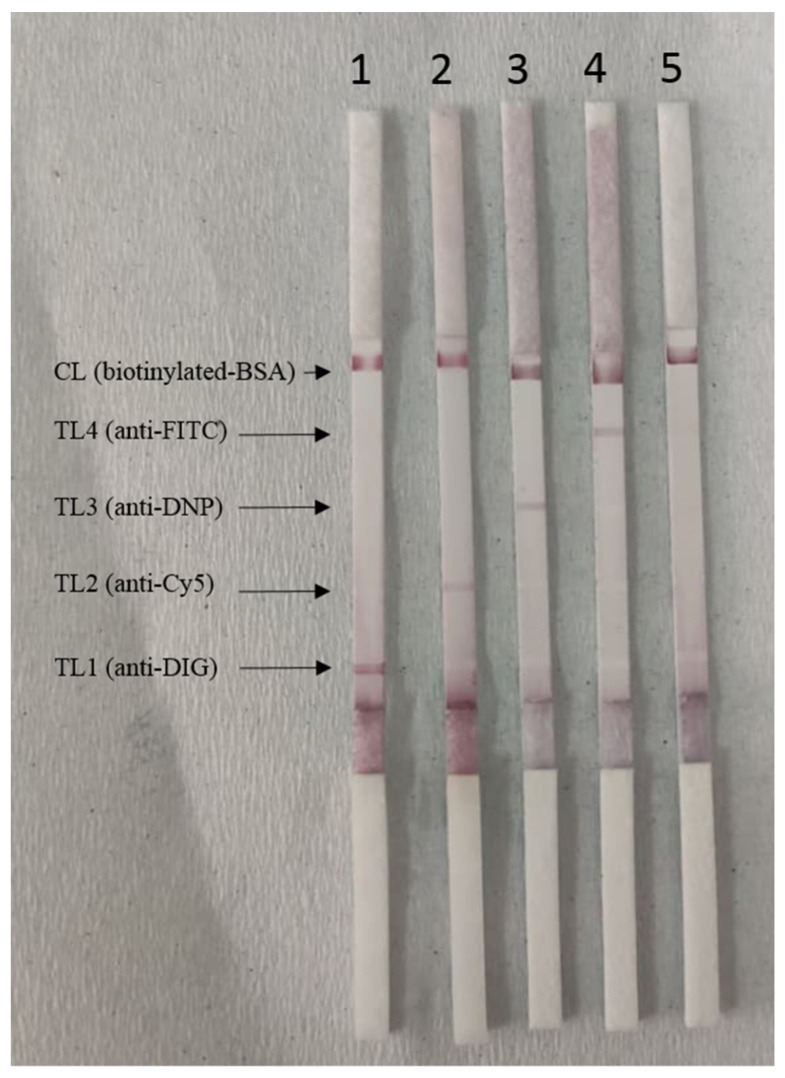
Singleplex testing of the LAMP-LF assay. Strip 1: *P. malariae*; Strip 2: *P. falciparum*; Strip 3: *P. vivax*; Strip 4: *P. knowlesi*; Strip 5: negative control (distilled water).

Figure 2 shows the results for the singplex LAMP-LF. Strip 1 shows a positive LAMP-LF result of *P. malariae* that appeared in two red bands, respectively located in the test line 1 (TL1) coated with anti-DIG and control line (CL) coated with biotinylated BSA. Strip 2 shows a positive LAMP-LF result of *P. falciparum* that appeared in two red bands, respectively located in the test line 2 (TL2) coated with anti-Cy5 and CL. Strip 3 shows a positive LAMP-LF result of *P. vivax* that appeared in two red bands, respectively located in the test line 3 (TL3) coated with anti-DNP and CL. Strip 4 shows a positive LAMP-LF result of *P. knowlesi* that appeared in two red bands, respectively located in the test line 4 (TL4) coated with anti-FITC and CL. Strip 5 shows the LAMP-LF result of the negative control (distilled water) that appeared only in the CL. Different positive LAMP products were tested on the lateral flow strips. There was no cross-reactivity between the positive LAMP products with different haptens and the antibodies coated at the lateral flow strips (figure not shown). The control line indicated the proper liquid flow through the strip.

**Figure 3:**
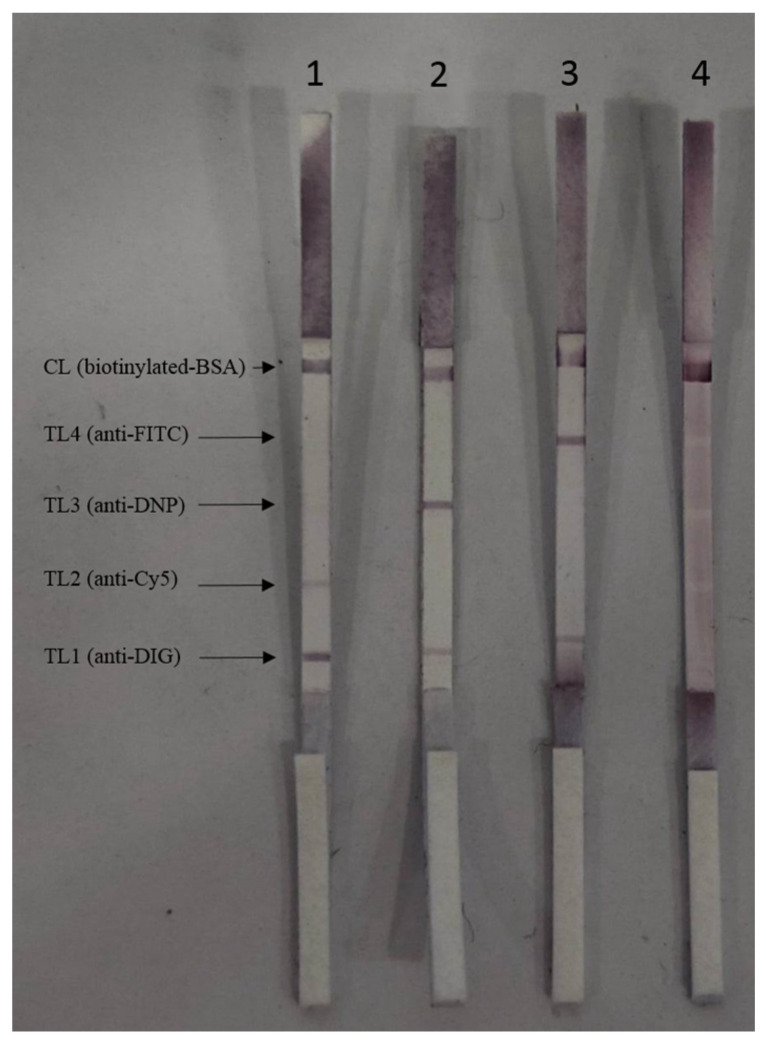
Representative multiplex LAMP-LF results for both archived and clinical samples. Strip 1: *P. falciparum*; Strip 2: *P. vivax*; Strip 3: *P. knowlesi*; Strip 4: negative control (distilled water).

Figure 3 shows the multiplex LAMP-LF results for both archived and clinical samples. Strip 1 shows a positive LAMP-LF result of *P. falciparum* that appeared in three red bands, located in the TL1 coated with anti-DIG, TL2 coated with anti-Cy5, and CL, respectively. Strip 2 shows a positive LAMP-LF result of *P. vivax* that appeared in three red bands, located in the TL1 coated with anti-DIG, TL3 coated with anti-DNP, and CL, respectively. Meanwhile, strip 3 shows a positive LAMP-LF result of *P. knowlesi* that appeared in three red strips, located in the TL1 coated with anti-DIG, TL4 coated with anti-FITC, and CL, respectively. Strip 4 shows the LAMP-LF result of the negative control (distilled water) that appeared only in the CL.

Table 2 shows the result of the comparison between microscopy and multiplex LAMP-LF for archived malaria patients’ samples. The multiplex LAMP-LF successfully amplified all *P. knowlesi* (n = 26), *P. falciparum* (and = 9), *P. vivax* (n = 9), *P. malariae* (n = 2), and *P. ovale* (n = 2) positive samples. The 20 healthy blood samples did not show any amplification. By using microscopy as the reference method, the multiplex LAMP-LF assay showed 100% sensitivity (95% Confidence Interval (CI): 92.6 to 100%) and 100% specificity (95% CI: 83.2 to 100%).

Table 3 shows the results of the comparison among microscopy, nested PCR, and multiplex LAMP-LF for the malaria patients’ samples collected from Hospital Kapit, Sarawak. The results show that there were two samples detected as negative by both microscopy and multiplex LAMP-LF but were detected as P. vivax and P. knowlesi, respectively by nested PCR. Also, another sample that was detected as P. knowlesi by both microscopy and multiplex LAMP-LF but negative by nested PCR. Besides, we found that one sample was detected as P. falciparum by microscopy but as P. knowlesi by both multiplex LAMP-LF and nested PCR.

By using microscopy as the reference method, the clinical sensitivity and specificity of multiplex LAMP-LF were calculated based on 86 samples collected on-site. With this, the developed multiplex LAMP-LF achieved 100% sensitivity (95% CI: 91.4 to 100%) and 97.8% specificity (95% CI: 88.2 to 99.9%). Multiplex LAMP-LF successfully amplified all microscopy-positive samples. The 44 healthy blood samples did not show any amplification. The positive predictive values and negative predictive values were 99.8% (95% CI: 99.2–99.95) and 100%, respectively. We calculated the kappa coefficient for all the methods using microscopy as a reference test. Our results show that nested PCR had 94.2% agreement (kappa = 0.88, 95% CI: 0.78–0.98), and the multiplex LAMP-LF 98.8% agreement (kappa = 0.98, 95% CI: 0.93–1).

## 4. Discussion

In this study, we have developed a multiplex LAMP-LF assay for the diagnosis of human malaria. The lateral flow strips contain test lines that capture biotin-, FITC, DIG-, Cy5-, and DNP-labelled amplicons. The overall process was completed in one hour. The developed multiplex LAMP-LF shows 100% clinical sensitivity and 97.8% clinical specificity for the clinical samples collected on-site. This is a rapid, highly sensitive, robust, and easy-to-operate method. Results can be read by observing the visible lines on the lateral flow strips without the need for special equipment. Multiplex LAMP-LF is suitable for use outside research/diagnosis laboratories and in field settings.

Out of the 86 samples, one sample was positive for *P. knowlesi* in both microscopy and LAMP-LF but negative when tested with nested PCR. Two microscopy and LAMP-LF negative samples were also found to be positive for *P. knowlesi* and *P. vivax*, respectively, when tested with nested PCR. Based on these results, we infer that the discrepancy may be due to the approaches used for DNA extraction between LAMP-LF and nested PCR in this study. When comparing the two extraction methods, the simple yet crude DNA extraction method used for LAMP-LF may have given a lower DNA yield compared with the conventional solid phase DNA extracted by a commercial DNeasy^®^ Blood & Tissue Kit (Qiagen, Hilden, Germany). Therefore, nested PCR was able to pick up the two negative samples that were undetected by LAMP-LF and microscopy. There was also one sample detected as *P. falciparum* by microscopy but detected as *P. knowlesi* by LAMP-LF and nested PCR. It may be due to the misdiagnosis of microscopy as the morphological features of the early trophozoites of *P. knowlesi* are similar to that of *P. falciparum* [30]. Due to the rarity of *P. ovale* and *P. malariae* samples, these species of malaria were not tested using this protocol.

The method used in DNA extraction was adapted from Zou et al. [20]. This is a simple and DNA purification-free protocol. According to the WHO, an ideal point-of-care diagnostic must fulfill the ASSURED criteria. LAMP, in itself, is an ideal candidate as a point-of-care diagnostic tool for human malaria as it complies with most of the criteria. Namely, LAMP is affordable; it is more sensitive and specific when compared with nested PCR as it involves 4 to 6 primers; it is user-friendly as it does not require expertise for species identification as in the case of microscopy; lastly, LAMP amplification is rapid and the assay can be completed in less than one hour. However, to use LAMP as a diagnostic approach, DNA is required as a template. Most of the DNA extraction methods require a centrifuge, commercial DNA extraction kits, and multiple liquid handling steps, which do not fit the criteria of point-of-care. However, the DNA extraction protocol developed here is fast, easy-to-use, and simple to handle. Without the need of a centrifuge, the overall extraction protocol took only ~8 min to complete.

Additionally, without a turbidity meter or gel electrophoresis machine, the LAMP endpoint readout cannot be properly ascertained. A colorimetric detection approach could be used to solve the problem. Additional colorimetric dyes in the LAMP assay could be used to determine between positive and negative results by observing the color change with the naked eye. However, the colorimetric detection approach is not suitable for use with samples with mixed infections. Therefore, we propose a multiplex LAMP-LF to solve this issue. For example, for samples with *P. knowlesi* and *P. falciparum* mixed infections, the red color bands of anti-FAM, anti-Cy5, anti-DIG, and control lines were visible on the lateral flow strip. The result was visualized within 5 min.

According to Zou et al. [20], filter papers can be made into dipsticks to extract nucleic acids from a wide range of biological samples in less than 30 s without the need for any special equipment. Blood was one of the biological samples tested. We adapted this method in this study for the diagnosis of malaria with some modifications. To assist the binding of DNA to the filter paper, we increased the size to a 6-mm diameter filter paper. We also made some amendments to the washing step. The volume of the washing buffer was increased to 1 mL instead of 200 μL as we found that a larger buffer volume may better help with diffusion of contaminants from the filter paper. When the filter paper was dipped in the LAMP reaction mixture, the captured DNA was slowly diffused from the filter paper into the LAMP reaction mixture and subsequently amplified.

LAMP-LF technology was used for the detection of malaria many years ago. However, most of their reports focused on single or duplex *Plasmodium* species detection. Sharma et al. developed a duplex LAMP-LF for the detection of *P. falciparum* and *P. vivax* [31]. Yongkiettrakul et al. also reported a LAMP-LF assay for the detection of *P. falciparum* and *P. vivax* by using dihydrofolate reductase thymidylate synthase (*dhfr-ts*) as the target gene [32]. Although the LAMP-LF was sensitive and could detect as little as 1 picogram (pg) of *P. falciparum* DNA and 1 nanogram (ng) of *P. vivax* DNA, the overall LAMP-LF assay took approximately 90 min to complete. As compared to our multiplex LAMP-LF developed here, the overall process only took 60 min to complete.

In 2018, Mallepaddi et al. reported a LAMP-LF for the detection of 5 *Plasmodium* species and successfully detected down to 0.01 pg/μL for all 5 *Plasmodium* species [33]. Although the entire LAMP-LF assay took a shorter time (approximately 42 min) to complete, only single species could be detected at each time of diagnosis. To improve the performance of LAMP-LF, we developed a multiplex LAMP-LF for the simultaneous detection of 5 *Plasmodium* species. Differentiation of the 5 *Plasmodium* species could be performed by using primers with differently labeled haptens. To the best of our knowledge, this is the first multiplex LAMP-LF simultaneously detecting the 5 *Plasmodium* species.

One of the limitations of this study was the comparison between tests was performed in a non-blinded fashion, which can lead to biases, particularly observer bias. The strength of this simple DNA extraction coupled with LAMP-LF developed in this study is its affordability, especially in areas with limited financial resources. The cost per reaction of LAMP-LF (USD 2.76) was slightly higher compared with nested PCR (USD 1.01), but with the advantage of not requiring a thermocycler and an electrophoresis apparatus. In this study, the cost per reaction of the simple DNA extraction (USD 0.11) was much lower compared with that of the commercial DNA extraction kit (USD 5.14).

## 5. Conclusions

In summary, we designed and tested a multiplex LAMP-LF on malaria that enables one to interpret the diagnostic results rapidly, precisely, and visually from clinical samples without professional instruments and expertise. The high sensitivity and specificity of multiplex the LAMP-LF developed here inspire confidence in this assay as a point-of-care method. By coupling a simple and purification-free DNA extraction method, the DNA extraction time can be reduced to ~ 8 min. This allows the diagnosis of malaria to be more accessible and affordable. It would be especially useful in resource-deficient areas.

## Figures and Tables

**Table 1:** LAMP-LF primers used in this study.

Species	Primer	Sequence 5’ to 3’
*Plasmodium* spp.	FIP	TACGGCCCGACGGTAAGATCGTAACCATGCCAACAC
	BIP	AGGAGTCTCACACTAGCGACAAAATTCCTTGTCGGGTAATCTC
	FLP	Biotin-CCGTCATAGCCATGTTAG
	BLP	DIG-ACCACATCTAAGGAAGGCAG
	F3	TGTCAACTACCATGTTACGAC
	B3	AACGGTCCTAAGGTAGCAA
*P. knowlesi*	FIP	GTTGTTGCCTTAAACTTCCTTGTGTTCTTGATTGTAAAGCTTCTTAGAGG
	BIP	TGATGTCCTTAGATGAACTAGGCTTTGCAAGCAGCTAAAATCGT
	FLP	Biotin-TAGACACACATCGTT
	BLP	FAM-GCACGCGTGCTACACT
	F3	CCATCTATTTCTTTTTTGCGTATG
	B3	CAGTGGAGGAAAAGTACGAA
*P. vivax*	FIP	GCCATGTTAGGCCAATACCCTAATGTGTGTATCAATCGAGTTTCT
	BIP	TAACGGGGAATTAGAGTTCGATTCCTGTAATTTACGCGCCTGCT
	FLP	Biotin-CATCAAAAGCTGATAGGTC
	BLP	DNP-GGAGAGGGAGCCTGAGAAATAGC
	F3	AGCGACACGTAATGGATC
	B3	CTTGTCACTACCTCTCTTCT
*P. falciparum*	FIP	AGTAGTCCGTCTCCAGAAAATCTTACTTTGGGGGCATTCGTATT
	BIP	GCGAAAGCATTTGCCTAATCTATTTAAGATTACGACGGTATCTGATC
	FLP	Biotin-TCACCTCTGACATCTG
	BLP	Cy5- GTTAAGGGAGTGAAGACG
	F3	GCTTAGTTACGATTAATAGGAGTA
	B3	AGTCGGCATAGTTTATGGT

FIP: Forward inner primer; BIP: backward inner primer; FLP: forward loop primer; BLP: backward loop primer; F3: forward primer; B3: backward primer.

**Table 2:** Comparison between microscopy and multiplex LAMP-LF for archived malaria patients’ samples.

Multiplex LAMP-LF	Microscopy						
	*Pf*	*Pk*	*Pv*	*Pm*	*Po*	Negative	No. Cases by Multiplex LAMP-LF
*Pf*	9	0	0	0	0	0	9
*Pk*	0	26	0	0	0	0	26
*Pv*	0	0	9	0	0	0	9
*Pm*	0	0	0	2	0	0	2
*Po*	0	0	0	0	2	0	2
Negative	0	0	0	0	0	20	20
Total	9	26	9	2	2	20	68

*Pf*: *P. falciparum*; *Pk*: *P. knowlesi*; *Pv*: *P. vivax*; *Pm*: *P. malariae*; *Po*: *P. ovale*.

**Table 3:** Comparison among microscopy, nested PCR, and multiplex LAMP-LF for the malaria patients’ samples collected from Hospital Kapit, Sarawak.

Samples		Microscopy	Nested PCR	Multiplex LAMP-LF
*P. knowlesi*	Positive	31 + ^β^1	^#^1 + *1 + 31	*1 + ^β^1 + 31
	Negative	0	0	0
*P. falciparum*	Positive	*1 + 2	2	2
	Negative	0	0	0
*P. vivax*	Positive	7	^α^1 + 7	7
	Negative	0	0	0
Negative	Positive	0	0	0
	Negative	^#^1 + ^α^1 + 42	42 + ^β^1	^#^1 + ^α^1 + 42

^#^ Sample was detected negative by microscopy and multiplex LAMP-LF but as *P. knowlesi* by nested PCR; ^α^ Sample was detected negative by microscopy and multiplex LAMP-LF but as *P. vivax* by nested PCR; ^β^ Sample was detected as *P. knowlesi* by microscopy and multiplex LAMP-LF but negative by nested PCR; * Sample was detected as *P. falciparum* by microscopy and *P. knowlesi* by multiplex LAMP-LF and nested PCR.

## Data Availability

The authors can confirm that all relevant data are included in the article.
